# Empathy reduces susceptibility to false memory

**DOI:** 10.1038/s41598-021-02281-4

**Published:** 2021-11-25

**Authors:** Shih-Yu Lo

**Affiliations:** 1 Institute of Communication Studies, National Yang Ming Chiao Tung University, 1001 University Road, Hsinchu, 300 Taiwan; 2 Institute of Communication Studies, National Chiao Tung University, Hsinchu, Taiwan

**Keywords:** Psychology, Human behaviour

## Abstract

Psychological and physiological evidence has demonstrated that the underlying mechanisms for empathy and for autobiographical memories were related to a great extent. However, whether the facilitative effect of empathy on memory also applied to misinformation was unknown. To test this, we used a misinformation paradigm on a sample of 51 participants aged 20–27. The participants viewed videos that evoked different degrees of empathy, and then were fed misleading information. The participants’ susceptibility to misleading information was lower for the videos that provoked a high degree of empathy compared to the videos that provoked a low degree of empathy. Based on our data, we conclude that empathy can prevent people from being misled by false information.

## Introduction

Episodic memories, which refer to the recollections of what we have done, experienced and felt, shape how we think we are. With episodic memories, we are able to maintain a coherent sense of self, so we still think that we are the same person even when our appearances and behaviors may have changed. The close relationships between the mental processes for episodic memories and those for the sense of the self have been corroborated by improved memory performance if one relates memories with themselves^1^, as well as by brain imaging evidence of a more extensive network of brain areas associated with self-related memories than self-unrelated memories^2^.

The self-superiority effect on memory can possibly be extended to memories about other people if one can relate themselves with others. The capacity to interpret the mental states of others is called “theory of mind” (ToM), and the ability to represent other people’s thoughts or feelings is referred to as empathy. Functional MRI studies have demonstrated that the brain regions supporting ToM and cognitive empathy overlapped with those underlying autobiographical memory^3–5^. Behavioral research also demonstrated a positive correlation between memory performance and empathy^6^. In the study of Ciaramelli et al.^6^, for example, the participants read fictitious life stories, followed by a memory task. The number of details they could recall from the stories positively correlated with how much they empathized with the characters.

### Empathy and memory

In the English language, the term “empathy” was first coined by Edward Titchener (1867–1927) in 1909 as a way to describe people’s ability to feel or understand what other people experience. *Dispositional* *empathy* is defined as a personality trait that varies between individuals^7^, while *situational* *empathy* can be triggered by external events^8,9^. In the memory task of the study of Ciaramelli et al.^6^, the life stories might have triggered the participants’ situational empathy, prompting them to put themselves in the shoes of the characters in the stories. The more they could take the first-person perspective, the more details they could remember.

Memory researchers have postulated that memory operates according to dual-process models. In such models, memory performance is determined by the participants’ *recollection* of or *familiarity* with a past event^10–12^. Recollection involves the retrieval of episodic information of an event one has encountered earlier, whereas familiarity refers to one’s general feeling of remembrance without being able to specify episodic or contextual details. The two types of memory processes could play different roles in identifying the source of a memory. For example, when you hear a story, the psychological process of source monitoring enables you to make a connection between the story and the storyteller. The process of recollection could contribute to correct source retrieval^13–15^, while the role of familiarity in source monitoring is ambivalent^16^. For example, when you hear multiple stories from multiple storytellers, recollection enables you to correctly associate stories with their tellers, whereas familiarity increases your generic familiarity for all the stories and the tellers without guaranteeing their correct match; when the numbers of the stories or the storytellers increase, mismatches are likely to occur.

In our daily life, we are bombarded with information from miscellaneous sources, and mismatches between memories and their sources lead to false memories. The way in which people are affected by false memories has been intensively examined by the misinformation paradigm^17,18^. This paradigm comprises three stages: in stage 1, the participant witnesses an event; in stage 2, some participants were fed misinformation; in stage 3, participants were instructed to recall or recognize the event in stage 1. Evidence has shown that people tend to recall the misinformation in stage 2 as true events occurring in stage 1^19–22^.

Research on the interactions between emotion and memory has shown that emotional information is retained more vividly than non-emotional information^23–26^, through a cascade of endogenous neurohormone activations^23,27–29^. However, the enhancement effect only applies to a small amount of contents that contains highly emotional information, leading to negligence of the rest^31,32^. A possible explanation is that when attention is drawn to the emotionally salient stimuli, fewer resources are available for processing other details^23,33^. For example, when people listen to an emotional story, they become deeply focused on specific emotional contents in the story, and, thus, their familiarity toward these contents increases; however, this might impede their ability to match the contents and the sources, leading to the formation of false memories^30^. In other words, emotion causes a central-peripheral trade-off effect, where the central information contains the emotionally arousing elements, and the peripheral information refers to the rest, including the source of the information.

How about empathy? A conventional way to induce emotions in previous studies is presenting the research partcipants with threatening objects or images, which were referred to as the “attention magnets^34^” as they could grab attention effectively. Emapthy, on the other hand, is induced by themantically arousing events instead of a particular object or an image. With this type of thematically arousing events, Laney and colleagues^34^ have demontrated that the enhancement effect of empathetic arousal on memory was more widepread. Based on this finding, we hypothesize that the presumably widespread effect of emapthy should also consolidate the connections between the memory contents and their sources, leading to a reduced likelihood of false memories, and an experiment was conducted to test this hypothesis.

### Research design

To test how empathy modulates false memories, we used an experimental paradigm similar to a study that probed the emotional effect on false memories developed by Van Damme and Smets^30^. The participants were presented with short videos that provoked different degrees of empathy, and then half of them were fed misleading information. The influence of misleading information on memories could be inferred by comparing the memory performance of the participants who were fed misinformation and the participants who were not.

Previous research on emotion has shown that emotion enhances the memory only for central information, while it does not affect or even suppress the memory for peripheral details^35,36^. In terms of how to experimentally manipulate the central versus peripheral quality of the information, Van Damme and Smets^30^ asked five people to draw lines around the areas that contained the source of emotion and define the central information as the information within the average area provided by the five judges. This method could potentially suffer from the judges’ subjectivity; furthermore, according to the study of Laney and colleagues^34^, emapthy is induced by themantically arousing events instead of a particular object or image that serves as the “attention magnet.” Therefore, we used a different way to distinguish central and peripheral information in the present study. The central information in the videos was defined as noticeable information and peripheral information as unnoticeable details. Thus, we designed a set of questions for each video and collected the participants’ response accuracy to these questions in a pre-test (Please refer to Pre-test 2 in the Method section). Central information was defined as the information in questions where participants could answer with a high accuracy, and peripheral information was defined as the information in questions where participants answered with a low accuracy.

A three-factor mixed-design experiment was conducted. The between-subject factor was Group, where half of the participants were fed misleading information and the other half was not. One within-subject factor was Empathy, for which each participant viewed a set of videos that provoked a high degree of empathy and another set of videos that provoked a low degree of empathy. The other within-subject factor was Difficulty, for which the participants had to answer one question regarding the central information (easy question) and another regarding the peripheral information (difficult question) for each video. For both of these questions, participants in the misleading group were fed misleading information, and, therefore, the participant’s responses in these two questions formed the index of false memories. In addition to two critical questions that were used to probe the formation of false memories, participants also had to answer four true-memory questions regarding true information in the videos, with a correct answer “yes” for two of them and a correct answer “no” for the other two.

### The filler task that serves a twofold purpose

To maintain an optimal difficulty level, we inserted a filler task between the memory acquisition phase and the test phase. This task served as a filler task; it was also endowed with another purpose: to test the extent to which empathy and self-representation are related. This was inspired by the finding of Sui, et al. ^37^, which concluded that people were able to relate external stimuli with themselves. Participants were instructed to associate themselves, a friend, or a stranger with three geometric patterns (a circle, triangle or square), and their perceptual judgments of the pattern representing themselves was better than those of the patterns representing others. Given empathy involves simulating or experiencing the feelings of others, people with a higher degree of empathy should perceive others to be less different than people with a lower degree of empathy. Therefore, it can be hypothesized that people with a higher degree of empathy should demonstrate the least degree of sensitivity to self-related stimuli compared to others. We used the same task and tested whether this self-superiority effect varied with the participants’ degree of empathy.

## Results

### Manipulation check

In the main experiment, we used six high empathy-inducing videos and six low empathy-inducing videos, based on a pre-test (Please refer to Pre-test 1 in the Method section) that measured the perceived empathy. To ensure that the two sets of videos differed only on the dimension of empathy, we deliberately chose the two sets that did not significantly differ on any dimension of emotion^38^, including positive valence, negative valence, or arousal. Positive valence represents a high degree of pleasure, whereas negative valence represents the opposite; the degree of arousal refers to the level of physical activation evoked by the emotional event.

However, the data in the main experiment still showed that the degree of negative valence (*F*[1,50] = 14.79, *p* < 0.001, *η*^*2*^_*partial*_ = 0.23) as well as arousal (*F*[1,50] = 26.31, *p* < 0.001, *η*^*2*^_*partial*_ = 0.34) was significantly higher for the high empathy-inducing videos than for low empathy-inducing videos. To fix this, we removed the most arousing video in the high-empathy group and the least arousing video in the low-empathy group. For the remaining 10 videos with five videos in each group, the ratings of perceived empathy were still significantly higher for the high empathy-inducing videos than for low empathy-inducing videos (*F*[1,50] = 42.67, *p* < 0.001, *η*^*2*^_*partial*_ = 0.46), but the ratings for positive valence (*F*[1,50] = 2.55, *p* = 0.12, *η*^*2*^_*partial*_ = 0.05) and negative valence (*F*[1,50] = 2.69, *p* = 0.11, *η*^*2*^_*partial*_ = 0.05) were not significantly different between the two sets of videos. Nevertheless, the effect on arousal still reached statistical significance (*F*[1,50] = 6.42, *p* = 0.01, *η*^*2*^_*partial*_ = 0.11), despite the much lower effect size (*η*^*2*^_*partial*_ = 0.11) compared to perceived empathy (*η*^*2*^_*partial*_ = 0.46). All the reported data below are based on the 10 remaining videos, and the possible confounding effect of arousal will be discussed.

For the remaining 10 videos, the difficulty indices (based on Pre-test 2 on another group of 41 participants; the details for Pre-test 2 are described in the Method section) for the critical easy questions did not significantly differ between the high empathy-inducing and low empathy-inducing videos (*t*[40] = 0.22, *p* = 0.83, *Cohen’s* *d* = 0.03). The same was true for the critical difficult questions (*t*[40] < 0.01, *p* > 0.99, *Cohen’s* *d* < 0.01). Therefore, if there was any effect of empathy on memory, the effect was not confounded with difficulty, as the questions we used to test the memory performance had equal levels of difficulty between the high empathy-inducing videos and low empathy-inducing videos.

### Memory performance

The misinformation effect could be indexed by the difference between the accuracy of the critical questions in the misleading group and that in the control group. The critical questions were two alternative-choice questions; one option was the correct answer, and the other was incorrect information only provided in the misleading group. Any accuracy reduction in the misleading group compared to the control group should be due to the misinformation that was provided only to the misleading group.

We used a mixed-design analysis of variance (ANOVA) with the factors of Group (between-subject: misleading versus control), Empathy (within-subject: high empathy versus low empathy), and Difficulty (within-subject: easy versus difficult) on the accuracy of the critical questions. There was a three-way interaction among the three factors (*F*[1, 49] = 4.36 *p* = 0.04, *η*^*2*^_*partial*_ = 0.08); thus, we separated the critical easy questions (central information) and critical difficult questions (peripheral information) into two different ANOVAs.

**Figure 1:**
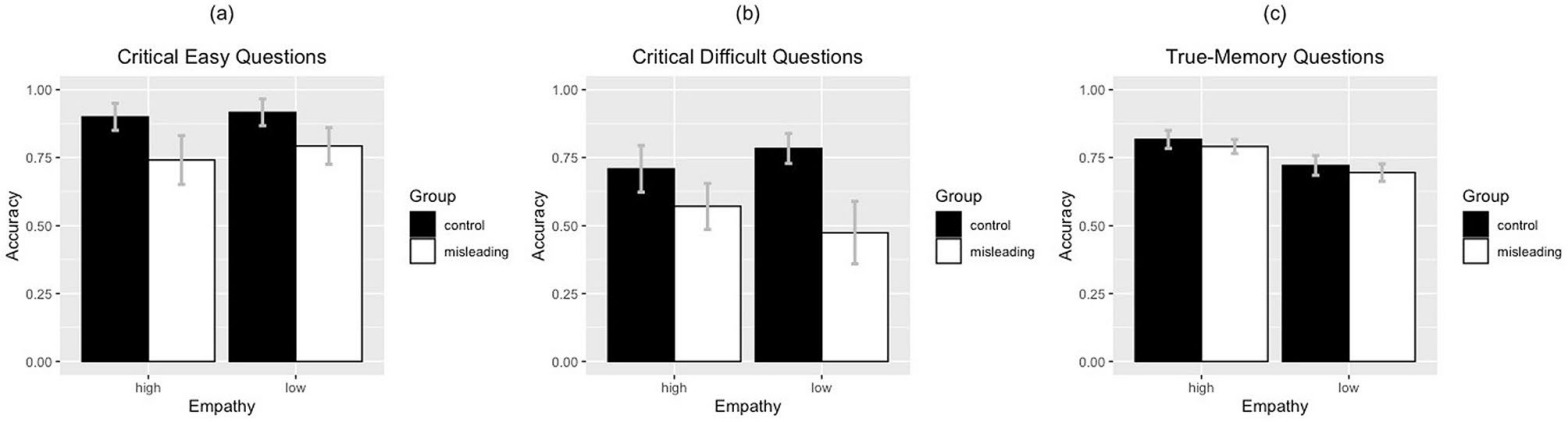
The accuracy of memory performance for **(a)** critical easy, **(b)** critical difficult, and **(c)** true-memory questions. The error bars indicate the 95% confidence interval.

For the critical easy question (Fig. 1a), Group had a significant effect (*F*[1, 49] = 16.48, *p* < 0.001, *η*^*2*^_*partial*_ = 0.25); the accuracy of the misleading group (*M* = 0.77) was lower than that of the control group (*M* = 0.91). Therefore, our manipulation on the misinformation was successful. However, empathy had no significant effect (*F*[1, 49] = 1.28, *p* = 0.26, *η*^*2*^_*partial*_ = 0.03), and there was no interaction between the two factors (*F*[1, 49] = 0.32, *p* = 0.58, *η*^*2*^_*partial*_ = 0.006).

For the critical difficult questions (Fig. 1b), there was a significant interaction between Group and Empathy (*F*[1, 49] = 4.06, *p* = 0.049, *η*^*2*^_*partial*_ = 0.08). According to the benchmarks provided by Cohen^39^ , this effect was considered to be between medium (*η*^*2*^ = 0.06) and large (*η*^*2*^ = 0.14). A simple main effect analysis showed that the misinformation effect, indicated by the decrease in accuracy in the misleading group as opposed to the control group, was significant for both the low empathy-inducing videos (*M*_*difference*_ = 0.31, *t*[49] = 4.82, *p* < 0.001, *Cohen’s* *d* = 1.35) and the high empathy-inducing videos (*M*_*difference*_ = 0.14, *t*[49] = 2.36, *p* = 0.02, *Cohen’s* *d* = 0.66). More importantly, the significant interaction between Group and Empathy indicated that the misinformation effect was larger for the former than the latter, which could be understood as follows. For the low empathy-inducing videos, there was a decrease of 0.31 in accuracy when comparing the misleading group with the control group. The corresponding decrease in accuracy was significantly smaller for the high empathy-inducing videos, reaching only 0.14. In other words, a high degree of empathy prevented participants from being misled. It should be noted that the difficulty indices for the critical questions did not differ between the high empathy-inducing and low empathy-inducing videos, based on a pre-test (Please refer to Pre-test 2 in the Method section). Therefore, the questions for the high empathy-inducing videos were not particularly easier, and a reasonable way to explain the lower susceptibility to misinformation for the high empathy-inducing videos is the provoked empathy.

For the true-memory questions (Fig. 1c), we averaged the accuracy for the two “yes” questions and two “no” questions to determine the overall accuracy for the veridical information in the videos. There was a significant effect for empathy (*F*[1, 49] = 57.78, *p* < 0.001, *η*^*2*^_*partial*_ = 0.54), with participants answering the questions for the high empathy-inducing videos (*M* = 0.80) more accurately than for the low empathy-inducing videos (*M* = 0.71). There was no significant effect of Group (*F*[1, 49] = 2.16, *p* = 0.15, *η*^*2*^_*partial*_ = 0.04) or interaction (*F*[1, 49] < 0.01, *p* = 0.99, *η*^*2*^_*partial*_ < 0.001). This echoes the previous finding that empathy could positively predict memory^6^. However, it was difficult to determine whether the better performance for true events in the high empathy-inducing videos was caused by empathy or whether the questions were easier, as we did not include the true-memory questions in the pre-test (Please refer to Pre-test 2 in the Method section). Therefore, in the following discussions, we focus on the effect of empathy on false memories.

### The role–shape matching performance

Participants also performed a role–shape matching task where they had to associate themselves, a friend, and a stranger with different geometrical patterns. An instruction was given to, for example, associate themselves with a disc, a friend with a square, and a stranger with a triangle. Then they were presented with a text—“you,” “friend,” or “stranger”—together with a shape—a disc, a triangle, or a square, and they had to decide whether the text and the shape matched according to the instruction.

We first computed the *d’* value, which was an index of the participants’ capacity to discriminate a matched role–shape pair from a non-matched one. There was a significant effect of Role (you/friend/stranger) on *d’* (*F*[2, 100] = 8.88, *p* < 0.001, *η*^*2*^_*partial*_ = 0.15). Post hoc analyses showed that significantly higher *d’* values were associated with the target pattern that represented “you” compared to “friend” (*t*[50] = 3.51, *p* = 0.001, Cohen’s *d* = 0.49) and “stranger” (*t*[50] = 3.57, *p* = 0.001, Cohen’s *d* = 0.50). There was no significant difference between “friend” and “stranger” (*t*[50] = 0.44, *p* = 0.66, Cohen’s *d* = 0.06).

We also analyzed reaction time using the factors of Role (you, friend, stranger) and Matching (correct role–shape matching versus incorrect role–shape matching). There was an interaction between the two factors (*F*[2, 100] = 78.61, *p* < 0.001, *η*^*2*^_*partial*_ = 0.61). We then separated the match and mismatch conditions and found that Role had a significant effect on the match condition (*F*[2, 100] = 102.9, *p* < 0.001, *η*^*2*^_*partial*_ = 0.67) but not the mismatch condition (*F*[2, 100] = 3.01, *p* = 0.054, *η*^*2*^_*partial*_ = 0.06). In the match condition, the post hoc analyses showed that there was a significant difference between the reaction time for the target pattern that represented “you” versus “friend” (*t*[50] = 11.97, *p* < 0.001, Cohen’s *d* = 1.68) and “you” versus “stranger” (*t*[50] = 11.97, *p* < 0.001, Cohen’s *d* = 1.68). However, there was no significant difference between “friend” and “stranger” (*t*[50] = 1.30, *p* = 0.2, Cohen’s *d* = 0.18). The results for both *d’* and the reaction time were consistent with the study of Sui, et al.^37^, in which the participants were required to imagine one of their best friends for the target role of “friend.” We skipped this step in our procedure and obtained consistent results. Therefore, we believe that this step was not crucial.

To test the relationship between dispositional empathy and performance on their role–shape matching task, we first computed the selfness index by computing the difference in the *d’* value and the reaction time between the trials with the target shape representing “you” and the trials with the target shape representing “friend” and “stranger”. Note that the index based on the reaction time was computed only from the matching trials. We then computed the correlation between the selfness indices and the rating results in IRI, which is a measure of dispositional empathy. Pearson’s *r* was −0.12 between the IRI and selfness based on the reaction time (*t*[49] = 0.86, *p* = 0.39) and was -0.05 based on *d’* (*t*[49] = 0.34, *p* = 0.73). Neither value reached statistical significance.

## Discussion

The results of the study show that empathy reduced participants’ susceptibility to misinformation but only for critical difficult questions and not critical easy ones. Critical easy questions concerned noticeable details in the videos, and these details were probably already firmly consolidated before the participants were fed misleading information, regardless of how much empathy the videos provoked. For critical difficult questions, on the other hand, more effort was required to complete the consolidation process that was still incomplete when the misinformation appeared. This enabled empathy to exert a facilitative effect.

To experimentally tease apart the effects of empathy and emotion, we deliberately chose two sets of videos that were supposed to provoke different degrees of empathy but equivalent degrees of valence and arousal, based on the data collected in a pre-test (Please refer to Pre-test 1 in the Method section). In the main test, both the degrees of positive valence and negative valence were fairly constant between the high empathy-inducing videos and the low empathy-inducing ones, while the degree of arousal was significantly higher for the former type of videos. From a methodological perspective, we could not fully rule out the possible confounding role of arousal on the effect of empathy. From an ecological perspective, empathy and arousal might be two inseparable psychological phenomena that always occur together.

Empathic arousal is an element of empathy that appeared early during human development^40^. For example, the fact that children cry when they hear other people do so is one of the earliest demonstrations of empathy in human development^41^. Similar phenomena can be observed in many non-human animal species as well^42^. According to the model proposed by Decety et al.^43,44^, affective arousal is an important element that contributes to the experience of empathy, mediated by the amygdala, the hypothalamus, and the orbitofrontal cortex. Consistent with such a view, some researchers even used the degree of arousal as an implicit index of empathy^45^. Therefore, the provocation of empathy would inevitably elicit an increase in arousal. Despite the interdependence between empathy and arousal, empathy can still be dissociated from the other dimension of emotion, the negative emotional valence, in terms of the effect on false memory. To clarify the relationship between empathy, negative emotional valence, and memory, the dual-process models of memory need to be discussed.

Based on the dual-process models^11,12^, memory performance could be determined by recollection and familiarity, which yield distinct predictions of the effect of misinformation. Recollection involves associating the memory contents with the contexts or the sources, preventing the memory from being overwritten by subsequent events; familiarity, on the other hand, involves increasing people’s overall familiarity for a particular set of objects or events, without linking them to the contexts or the sources.

The emotional effect on memory has been widely investigated. In general, negative emotion enhances the encoding for both veridical and misleading information. Emotionally salient stimuli serve as the “attention magnets” to attract attentional resources, leaving fewer resources to peripheral details^23,33^. Consequently, people’s familiarity towards the “attention magnets” increases, leaving fewer resources to associate the emotional contents with their sources, causing false memories. In contrast, empathy is not induced by a small set of attention magnets that could attract attentional resources only to a small amount of objects or images. Furthermore, when empathy is provoked, the psychological mechanisms underlying autobiographical memory are triggered as well^4,5,46^, which strengthen the recollection process and consolidate the connections between memory contents and their sources. As a result, the provocation of empathy prevents the memory contents from being overwritten.

The association between empathy and autobiographical memory implies a connection between empathy and the representation of the self. The idea that the representation of the self can be modulated by empathy was tested in the study of Batson, et al.^47^ using the Inclusion of Other in the Self (IOS) scale^48,49^. Participants were presented with two circles with varying degrees of overlap. One of the two circles represented themselves and the other circle represented other people. Batson, et al.^47^ found that the participants in whom empathy was induced tended to choose circles with more overlap, compared to the neutral group. A possible explanation is that, with a higher degree of empathy, the representation of the self and that of others can be more connected.

In the present study, the role–shape association task not only served as a filler task but also endowed with another purpose to test whether the tendency to connect the representation of the self and that of others correlated with the trait of empathy. In our study, we found a significant improvement in perceptual judgment for shapes that were associated with the self, replicating the results of Sui, et al.^37^. However, the degree of improvement in performance correlated negligibly with the participants’ dispositional empathy measured by IRI. One possibility is that the self–other connectedness applied only to situational empathy, as in the case of the study of Batson, et al.^47^, and not to dispositional empathy, as in the case of this study. Another possibility is that the reaction time and the accuracy measurements used in the present study were not sensitive to manifest the representation of the self. Future studies, perhaps by means of neuroimaging methods, are required to test how empathy shapes the representation of the self.

Investigations into false memory have been pioneered by Loftus for over 30 years^18^. This topic has become even more relevant in the recent years due to the rapid development of social media, as information—including misinformation—can spread across borders within seconds. The impact of misinformation can be quite damaging, particularly during global crises. At the time this manuscript was being prepared, the world was suffering from the COVID-19 pandemic, and a recent study shows that belief in ungrounded conspiracy theories could potentially hinder people’s intentions to take prevention measures^50^. Empathy may be one of the psychosocial factors that can help alleviate the impact of misinformation. Empathy promotes pro-social behaviors^51^ while also preventing people from being misled by false information, as suggested by the data of the present study.

## Method

### Participants

The experimental protocol in this study was approved by the Research Ethics Committee for Human Subject Protection of National Yang Ming Chiao Tung University (Case number: NCTU-REC-108-001E), and all methods were performed in accordance with the relevant guidelines and regulations laid down by the ethics committee. Informed consent was obtained from all the participants.

According to Van Damme and Smets^30^, the effect size (*η*^2^) for how misleading information affected memory performance was 0.11 for peripheral details. Based on this effect size, we used G*Power 3^52^ to compute the required sample size for the present study. We set the test family as “F test,” the statistical test as “ANOVA: Repeated measures, between factors,” the number of groups as two, and the number of measurements as two. To achieve a power of 0.8, the sample size was estimated to be at least 50. We then recruited 51 participants (21 males) aged 20–27 (*M* = 22, *SD* = 2.03) based on convenience sampling. All the participants had normal or correct-to-normal vision and reported no psychopathological or neurological disorders.

### Procedure

We conducted two pre-tests prior to the main test. Pre-test 1 served to find usable videos to trigger different degrees of empathy, and Pre-test 2 served to find questions that were suitable for eliciting false memories. It should be noted that the participants in Pre-test 1, Pre-test 2, and the main test were different. The participant characteristics mentioned in the Participant section applied only to the main test, because our conclusions were based only on the main test; pre-tests 1 and 2 were only used to find out appropriate materials for the main test.

#### Pre-test 1

To collect experimental materials, we followed a procedure similar to that of the Socio-affective Video Task (SoVT) used by Klimecki, et al.^53^. We first collected short videos from documentaries, movie trailers, or TV shows. We assumed that, in most cases, videos that can provoke a higher degree of empathy are most likely to be those with a negative valence and high levels of arousal. Thus, we collected 110 videos that possessed these characteristics. In addition, we collected 10 videos that possessed a positive valence and high levels of arousal, 10 videos that possessed a positive valence and low levels of arousal, and 10 videos that possessed a negative valence and low levels of arousal. The duration of each video was approximately 20 s.

We then embedded these videos in an online questionnaire on the Qualtrics platform. We asked a group of 731 participants to provide ratings of positive valence, negative valence, arousal and empathy on the 11-point Likert scale (0 to 10) via the following questions: (1) *How* *much* *positive* *emotion* *did* *you* *feel* *in* *regard* *to* *this* *video?* (2) *How* *much* *negative* *emotion* *did* *you* *feel* *in* *regard* *to* *this* *video?* (3) *How* *aroused* *did* *you* *feel* *in* *regard* *to* *this* *video?* (4) *How* *much* *did* *you* *empathize* *with* *the* *people* *in* *the* *video?* Each participant had to fill out the ratings for 40 videos that were randomly selected from our set of 140. Based on the participant’s ratings, we selected six videos that could provoke high degrees of empathy and another six videos that could only provoke a low degree of empathy, while the two sets of videos induced the same degrees of positive emotion, negative emotion, and arousal. The difference in empathy ratings between these two sets of videos reached statistical significance (*t*[10] = 10.96, *p* < 0.001, *Cohen’s* *d* = 6.33). In terms of positive emotion (*t*[10]  = 0.26, *p* = 0.80, *Cohen’s* *d* = 0.15), negative emotion (*t*[10]  = 1.36, *p* = 0.20, *Cohen’s* *d* = 0.78), and degree of arousal (*t*[10]  = 0.22, *p* = 0.83, *Cohen’s* *d* = 0.13), the two sets of videos did not differ significantly.

#### Pre-test 2

For each of the 12 selected videos, we designed four misleading questions. For example, one video was the movie trailer of “Wonder,” depicting a boy who suffered from a medical facial deformity. The boy mentioned that he was 10 years old. We then designed a question, “How old was the boy in the video?” and provided the options of “ten” and “eight.” This served as Pre-test 2. We used Qualtrics to collect data from a group of 41 people. The error rate for each misleading question was used as an index of *difficulty* of this question. The error rate for the question presented above was 0.39, indicating that the likelihood for the participants to mistakenly answer “eight” was 0.39.

Of the four misleading questions developed for each video, we chose one with a high level of difficulty and labeled it as a difficult question and then chose one with a low level of difficulty and labeled it as an easy question. The difficulty level for the easy questions did not significantly differ between the high empathy-inducing and low empathy-inducing videos (*t*[40] = 0.21, *p* = 0.84, *Cohen’s* *d* = 0.03). The same was true for the difficult questions (*t*[40] = 0.98, *p* = 0.34, *Cohen’s* *d* = 0.15).

#### Main test

After signing the consent form, the participants filled out the questionnaire of the Interpersonal Reactivity Index (IRI)^54^, which measured their dispositional empathy. Then they were guided to a computer to conduct the experimental procedure.

**Figure 2:**
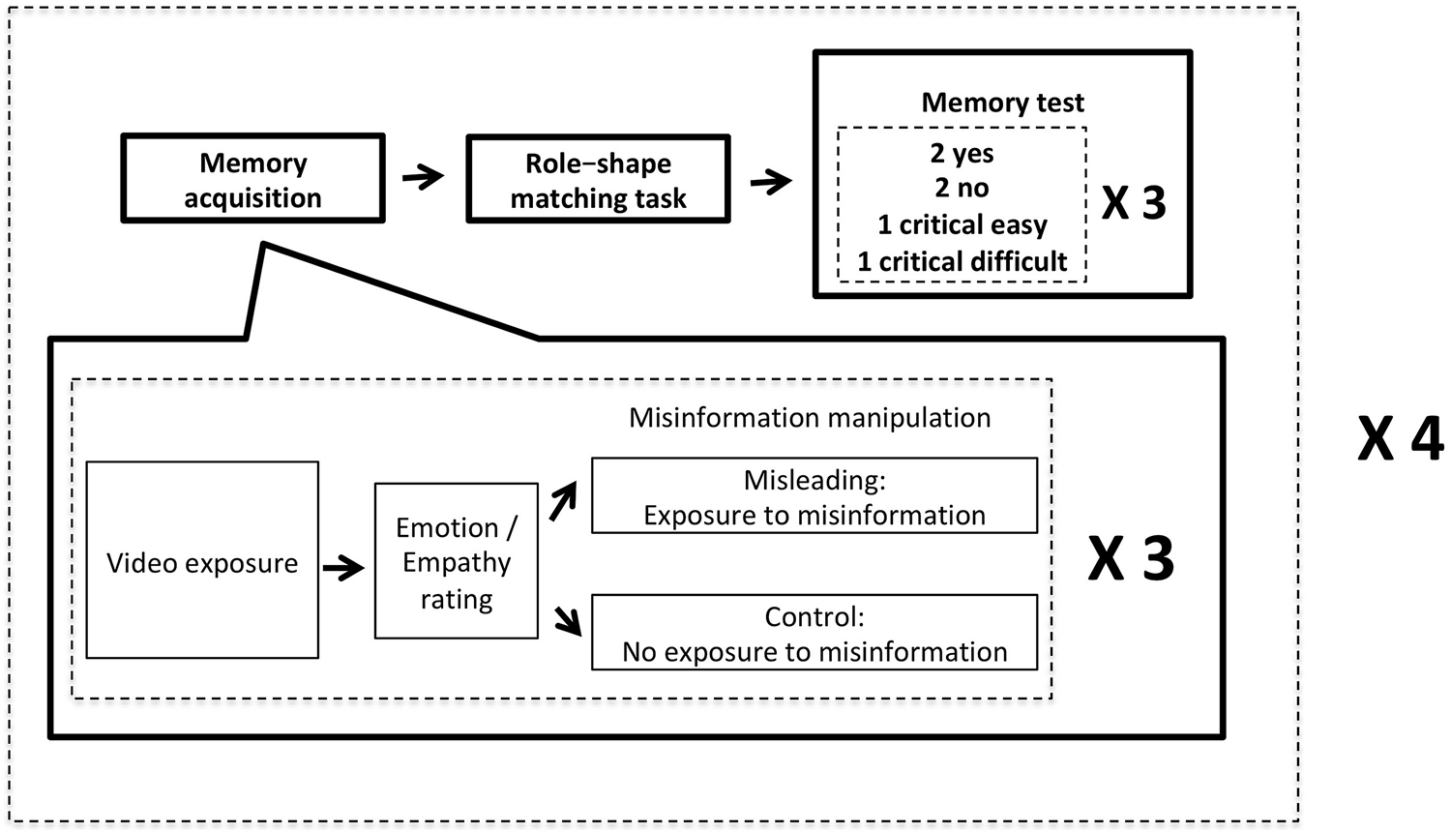
The structure of the experimental procedure. After filling out the IRI scale, the participants started with the memory acquisition stage, where they watched a video, summarized its contents, and rated the emotion and empathy indices about the video. In the misinformation manipulation stage, half of the participants were fed misinformation whereas the other half was not. After three cycles of video exposure, emotion/empathy rating, and misinformation manipulation for three videos, participants proceeded to the role–shape matching task, followed by the memory test stage, where they answered six questions for each video. The aforementioned procedure was repeated four times; therefore, each participant viewed 12 videos to complete the experimental session.

For a visualization of the procedure, please refer to Fig. 2. The participants were randomly assigned to the *misleading* (*n*_*m*_ = 27) and *control* (*n*_*c*_ = 24) group. Both groups started with the memory acquisition stage by watching a short video. In order to ensure that they had paid attention, they were asked to orally summarize its content. Then they had to rate four questions in the emotion/empathy rating stage on an 11-point Likert scale (0 to 10): (1) *How* *much* *positive* *emotion* *did* *you* *feel* *in* *regard* *to* *this* *video?* (2) *How* *much* *negative* *emotion* *did* *you* *feel* *in* *regard* *to* *this* *video?* (3) *How* *aroused* *did* *you* *feel* *in* *regard* *to* *this* *video?* *(4)* *How* *much* *did* *you* *empathize* *with* *the* *people* *in* *the* *video?*

After the emotion/empathy rating stage came the misinformation manipulation stage, in which the participants had to answer two questions that fed them misleading information. The two questions were modified from those used in Pre-test 2. For example, the original misleading question in Pre-test 2, *“How* *old* *was* *the* *boy* *in* *the* *video?* *(10/8),”* was modified to *“Do* *you* *remember* *how* *many* *surgeries* *the* *8-year-old* *boy* *had?* *(yes/no)”*. The two-alternative forced choice form in Pre-test 2 was modified into a yes/no form to insinuate participants with misinformation. For the control group, the misleading information was removed from the questions; for example, they were asked, *“Do* *you* *remember* *how* *many* *surgeries* *the* *boy* *had?* *(yes/no)”*. Four misleading questions were associated with each video in Pre-test 2, but only two were chosen for each video in the main test: one with a high level of difficulty and one with a low level of difficulty.

After completing three cycles of video exposure, emotion/empathy rating and misinformation manipulation, the participant was directed to another computer to complete the role–shape matching task, which served two purposes. First, it served as a filler task to separate the memory-acquisition stage and the memory-test stage, so the difficulty level of the memory task could be optimal; second, based on the previous study that showed a better identification performance for self-related shapes than other related shapes^37^, we examined the relationship between empathy and self-representation by measuring how this self-superiority effect co-varied with the dispositional empathy.

**Figure 3:**
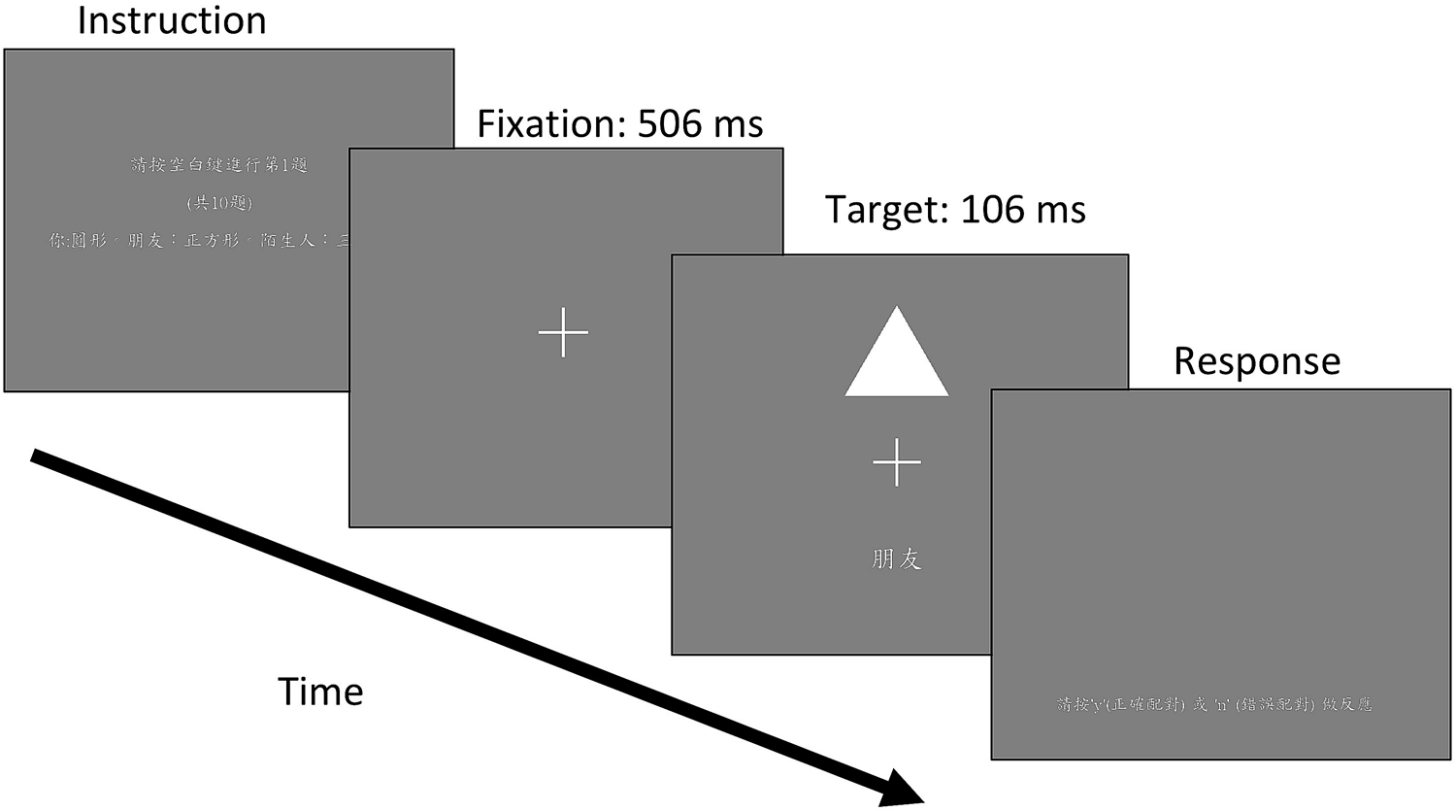
The stimulus sequence of the role–shape matching task. The text in the instruction display is translated as “*Please* *press* *space* *bar* *to* *proceed* *to* *Trial* *1*,” *“(10* *trials* *in* *total),”* and “*You:* *Disc;* *Friend:* *Square;* *Stranger:* *Triangle.”* The word shown in this example target display is “friend.” In the response display, the message can be translated as “please press ‘y’ (correct) or ‘n’ (incorrect) to respond.” In the actual experiment, there were 288 trials in total, and the response display was followed by a feedback display where “correct” or “incorrect” was printed at the center of the display. The “incorrect” feedback display was accompanied with a tone.

The role–shape matching task was programmed in MATLAB r2014b with Psychtoolbox-3 extensions^55,56^. Visual stimuli were displayed on a 17-inch CRT monitor (Mitsubishi i-TECH IF700 CRT monitor) with a spatial resolution of 1024 × 768 pixels and a refresh rate of 85 Hz. A viewing distance of approximately 65 cm was maintained. The participants were instructed to associate a shape with a role. One-third of the participants associated “you” with a disc, “friend” with a square, and “stranger” with a triangle. Another third of participants associated “you” with a triangle, “friend” with a disc, and “stranger” with a square. The final third associated “you” with a square, “friend” with a triangle, and “stranger” with a disc. The task started with 10 practice trials. Each trial began with an instruction display telling participants that they must press the space bar to begin, how many trials they had completed, and the matching rules for the task (Fig. 3). Then the fixation display—a white cross in which each arm had 1.76 degrees of visual angle—was presented in the middle of a grey background for 506 ms. This was followed by the target display for 106 ms. In this display, the fixation cross was in the middle and a geometrical pattern was placed 3.5 degrees above the center of the screen. The pattern could be a square with sides of 3.8 degrees of visual angle, a triangle with sides of 3.8 degrees of visual angle, or a disc with a diameter of 3.8 degrees of visual angle. The words “you,” “friend,” or “stranger” (written in Chinese) were placed 3.5 degrees below the center of the screen. Next came the response display, which told participants to press the “y” key to indicate correct matching or the “n” key to indicate incorrect matching. Following this, the feedback display was presented for 506 ms. If the participant gave a correct response within two seconds, “correct” was shown in the middle of the screen. If the participant gave an incorrect response within two seconds, “incorrect” was shown in the middle of the screen and an 800 Hz tone was played for 100 ms. If the participant did not make any response within two seconds, a message reminding them of the two-second maximum reaction time was shown in the middle of the screen with an 800 Hz tone played for 100 ms. Then came the instruction display for the next trial. The participants pressed any key to initiate the next trial. For a visualization of this procedure, please refer to Fig. 3.

After 10 practice trials, the participants began the main task. They completed 72 trials at their own pace and were then shown a display telling them to stop. Next, they were directed to the questionnaire computer (Fig. 2) to answer six questions for each of the three videos they had watched prior to the role–shape matching task. Among the six questions, two were descriptions that had been provided in the video, so the correct answer was “yes”; two were descriptions that had not been provided in the video, so the correct answer was “no”; the remaining two were critical questions, which were the same as the misleading questions in Pre-test 2 (e.g., *“How* *old* *was* *the* *boy* *in* *the* *video?* *[10/8]”*). One of the two critical questions was the easy question (low level of difficulty), and the other was difficult (high level of difficulty). The six questions marked the end of one cycle (memory acquisition, role–shape matching, memory test).

Each participant had to complete four cycles. For half of the participants, the first two cycles included videos that provoked a high degree of empathy, and the last two included videos that provoked a low degree of empathy. For the other half, the order was reversed. Therefore, the presentation order of the high empathy-inducing videos and the low empathy-inducing videos was counterbalanced. The descriptions about the videos and the questions used in the experiment are provided in the Appendix.

It should also be noted that the 288 trials (72 per cycle) of the role–shape matching task contained 96 trials for each shape (disc, triangle, or square). Each shape was paired with the matching role (me, friend, or stranger) in 48 trials and non-matching roles in 48 trials (24 for each of the other two). The trials of the different conditions were randomized for each participant.

### Data analysis for the role–shape matching task

The index of the participants’ capacity to discriminate a matched role–shape pair from a non-matched one, *d’*, was calculated based on the hit rate, which referred to the proportion of correct responses for the matching trials, and the false alarm rate, which referred to the proportion of incorrect responses for non-matching trials, according to the following formula:

$$d^{\prime} = \Phi^{ - 1} \left( H \right) \, {-}\Phi^{ - 1} \left( F \right)$$

The inverse Gaussian function, *Φ*^*−*1^, converts the hit rate (*Φ*^*−*1^[H]) and the false alarm rate (*Φ*^*−*1^ [F]) into *z* scores. To avoid infinite numbers, any hit rate or false alarm rate of 100% or 0 was adjusted respectively^57^ to (n − 0.5)/n or 0.5/n, where n (= 48) was the number of trials in each condition in the present study.

## Supplementary Information

Supplementary Information.

## Data Availability

The datasets generated and/or analyzed during the current study are available from the corresponding author on reasonable request.
