# Supplementary material for: Empathy reduces susceptibility to false memory
Source: Sci Rep. 2021 Nov 25;11:22903. doi: 10.1038/s41598-021-02281-4 (PMC8616898; doi:10.1038/s41598-021-02281-4)
Supplement: Supplementary file 1 — Supplementary Information. [file 41598_2021_2281_MOESM1_ESM.pdf]

Appendix: Videos and questions used in the experiment

*The decriptions with underlines are misleading information.

High empathy video 1

**Description**	
	A lady was sent overseas as a comfort woman during War World Two. She talked about how she was despised by her family members.
**Misinformation feeding**	
**Easy**	
Misleading	Was the senior lady in the video sent to Japan at the age of <u>22</u>? (yes/no)
Control	Was the senior lady in the video sent to Japan when she was young? (yes/no)
**Hard**	
Misleading	Was the lady in the video wearing a <u>blue</u> hat? (yes/no)
Control	Was the lady in the video wearing a hat? (yes/no)
**Questions**	
Critical easy	When was the lady in the film sent away? (22/19)
Critical hard	Was was the colour of the hat the lady in the video was wearing? (blue/black)
Yes	Did the lady in the video speak Taiwanese?
Yes	Did the lady in the video get kicked away by her family?
No	Is the surname of the lady in the video Huang?
No	Did the family also throw away the lady's wardrobe?

High empathy video 2

**Description**	
	A doctor in Yeman was talking about how the medical resources are lacking, and children died of starvation.
**Misinformation feeding**	
**Easy**	
Misleading	Do you remember in which city in <u>Oman</u> the video was shot? (yes/no)
Control	Do you remember in which city the video was shot? (yes/no)
**Hard**	
Misleading	Do you remember how old the doctor with a <u>mask</u> in the video was? (yes/no)
Control	Do you remember how old the doctor in the video was? (yes/no)
**Questions**	
Critical easy	Where was the video shot? (Oman/Yeman)
Critical hard	Was the female doctor in the video wearing a mask? (yes/no)
Yes	Was the baby in the video crying?
Yes	Was the hospital in the video named Sanaa?
No	Was the baby in the video wearing blue clothes?
No	Did the video mention that the Children died of human trafficking?

High empathy video 3

**Description**	
	The movie trailer of "Wonder"(Chinese title: The miraculous boy), which was about how a boy with facial deformity fitted in and coped with the difficulties in life.
**Misinformation feeding**	
**Easy**	
Misleading	Do you remember when the movie titled "the impossible boy" was released? (yes/no)
Control	Do you remember when the movie was released? (yes/no)
**Hard**	
Misleading	Do you remember how many surgeries the 8-year-old boy had? (yes/no)
Control	Do you remember how many surgeries the boy had? (yes/no)
**Questions**	
Critical easy	What was the title of the movie? (The impossible boy/The miraculous boy)
Critical hard	How old was the boy in the video? (8/10)
Yes	Was the movie released on Nov. 17?
Yes	Did the video mention that the boy had 27 surgeries?
No	Did the boy in the movie have red hair?
No	Was the boy in the video ridiculed by other girls?

High empathy video 4 (removed in data analysis)

**Description**	
	A girl was sobbing and talking about how she was physically abused by her mother.
**Misinformation feeding**	
**Easy**	
Misleading	Do you have any friend who is in a similar situation as the girl named "Summer" in the video? (yes/no)
Control	Do you have any friend who is in a similar situation as the girl in the video? (yes/no)
**Hard**	
Misleading	
	Do you remember the colour of the sweater the girl wore in the video? (yes/no)
Control	
	Do you remember the colour of the clothes the girl wore in the video? (yes/no)
**Questions**	
Critical easy	What was the name of the girl in the video? (Summer/Jasmine)
Critical hard	What was the girl in the video wearing? (sweater/shirt)
Yes	Was the girl in the video beaten by her mother?
Yes	Was the girl in the video sobbing?
No	Did the girl in the movie have a fringe?
No	Was the girl in the video bleeding?

High empathy video 5

**Description**	
	A documentary about teenage girls in Thailand who made their living by prostitution.
**Misinformation feeding**	
**Easy**	
Misleading	Do you remember what the girl with sunglasses said in the video? (yes/no)
Control	Do you remember what the girl said in the video? (yes/no)
**Hard**	
Misleading	Do you remember how many girls there were in the massage parlor? (yes/no)
Control	Do you remember how many girls there were? (yes/no)
**Questions**	
Critical easy	Was the girl being interviewed wearing sunglasses? (yes/no)
Critical hard	Where did the girls in the video stand? (massage parlor/karaoke place)
Yes	Was the documentary from CNN?
Yes	Was the documentary about teenage prostitution?
No	Was the city mentioned in the documentary Chiangmai?
No	Did the girl in the video suffer from domestic violence?

High empathy video 6

**Description**	
	A man with disability mentioned about how difficult to find a job.
**Misinformation feeding**	
**Easy**	
Misleading	Did Mr. Chou have a disability in his left leg? (yes/no)
Control	Did the man in the video have a disability in his left leg? (yes/no)
**Hard**	
Misleading	Had the man in the video consulted the employment service in Banchiao? (yes/no)
Control	Had the man in the video consulted any employment service? (yes/no)
**Questions**	
Critical easy	What is the surname of the man in the video? (Chou/Hsu)
Critical hard	Where was the employment service? (Banchiao/Shuling)
Yes	Did the man in the video ride a scooter?
Yes	Was the man in the video using a crutch?
No	Was the man in the video driving a car?
No	Was the man in the video wearing glasses?

Low empathy video 1

**Description**	
	A girl was talking to a crowd about how she was misunderstood and attempted to jump into a river.
**Misinformation feeding**	
**Easy**	
Misleading	Do you remember what the crowd shouted about with a megaphone? (yes/no)
Control	Do you remember what the crowd shouted about ? (yes/no)
**Hard**	
Misleading	Do you remember what the girl with a cleft lip talked about? (yes/no)
Control	Do you remember what the girl talked about? (yes/no)
**Questions**	
Critical easy	Did the crowd use a megaphone? (yes/no)
Critical hard	Did the girl have a cleft lip? (yes/no)
Yes	Was the girl in the video named "Yi-Yau"?
Yes	Was the girl in the video attempting to jump into a river?
No	Was it raining when the girl was running?
No	Did the girl in the video wear a coat?

Low empathy video 2

**Description**	
	A movie trailer about mysterious murder cases in a small American town.
**Misinformation feeding**	
**Easy**	
Misleading	Do you remember that in the video, it was mentioned that the small town was splitted into two parts, a girl named Lucy and a group of evil people? (yes/no)
Control	Do you remember that in the video, it was mentioned that the small town was splitted into two parts, a girl and a group of evil people? (yes/no)
**Hard**	
Misleading	Do you remember that the photo in the Instagram dialog box was a woman? (yes/no)
Control	Do you remember that the photo in the dialog box was a woman? (yes/no)
**Questions**	
Critical easy	The video mentioned about that the small town was split into two parts, a girl and a group of evil people. What was the name of the girl? (Lucy/Daisy)
Critical hard	A dialog box appeared in the video. What was the platform of the dialog box? (Instagram/Facebook)
Yes	Did the video mention that the grandson of a politician was released?
Yes	Did the video mention that a woman was lying in the yard?
No	At the end of the video, a person wore a mask. Was it a mask from the thriller movie "Saw"?
No	Was the photo in the dialog box a man?

Low empathy video 3

**Description**	
	A TV series trailer about the generation gaps between teenagers and their parents.
**Misinformation feeding**	
**Easy**	
Misleading	Do you remember the emotion state of the girl who was expected to go to a good high school? (yes/no)
Control	Do you remember the emotion state of the girll? (yes/no)
**Hard**	
Misleading	Do you remember that the boy was crying at the street corner? (yes/no)
Control	Do you remember that the boy was crying? (yes/no)
**Questions**	
Critical easy	The girl in the video was expected to go to a good: (high school / university)
Critical hard	The boy in the video was crying: (at the street corner / in bed)
Yes	Was the girl in the video wearing pink?
Yes	Did the girl in the video have a ponytail?
No	Were the two teenagers complaining about their fathers?
No	Did the boy wear glasses?

Low empathy video 4

**Description**	
	A TV series clip about a mother running into a movie theatre to find her son who was potentaiily killed.
**Misinformation feeding**	
**Easy**	
Misleading	Do you remember that the mother was held back by a female police officer when she was running into the movie theatre? (yes/no)
Control	Do you remember that the mother was held back by a woman when she was running into the movie theatre? (yes/no)
**Hard**	
Misleading	Do you remember that the mother's brown purse got pulled by the crowd? (yes/no)
Control	Do you remember that the mother's purse got pulled by the crowd? (yes/no)
**Questions**	
Critical easy	When the mother running into the movie theatre, she was held back by a: (police officer/ theatre staff member)
Critical hard	What was the colour of mother's purse? (brown/black)
Yes	Did the mother look back when she just entered the movie theatre?
Yes	Did the mother carry a phone when she entered the movie theatre
No	Did the mother carry sunglasses?
No	Did the mother wear a necklace?

Low empathy video 5 (removed in data analysis)

**Description**	
	A documentary about the poverty problems in a small mountainous village in China.
**Misinformation feeding**	
**Easy**	
Misleading	Do you remember what the boy was talking about while he had tears on his face? (yes/no)
Control	Do you remember what the boy was talking about? (yes/no)
**Hard**	
Misleading	Do you remember that in the end of the video, a boy was singing with laughters? (yes/no)
Control	Do you remember that in the end of the video, a boy was singing? (yes/no)
**Questions**	
Critical easy	Did the talking boy have tears on his face? (yes/no)
Critical hard	Did the singing come with laughters? (yes/no)
Yes	Was the village in the video a mountainous village?
Yes	Was the documentary shot in China?
No	Did the smoking boy have a scar on his face?
No	Was there any adult in the video?

Low empathy video 6

**Description**	
	A female sex worker was complaing about how she was ambushed by the police, while what she wanted was just to earn enough money for her to be financially independent when she got old.
**Misinformation feeding**	
**Easy**	
Misleading	Do you remember how old the interviewee who called herself "Peach" was? (yes/no)
Control	Do you remember how old the interviewee was? (yes/no)
**Hard**	
Misleading	Do you remember how the interviewee who had been a sex worker for 20 years was found by the authority? (yes/no)
Control	Do you remember how the interviewee was found by the authority? (yes/no)
**Questions**	
Critical easy	How did the interviewee call herself? (Peach/Apple)
Critical hard	How long had the interviewee been working as a sex worker? (20 years/30 years)
Yes	Was the interviewee complaining about the police?
Yes	Was the interviewee in the video in her 50s?
No	Was the colour of the interviewee red?
No	Did the interview speak Taiwanese?
